# Brief multifamily Psychoeducation for family members of patients with chronic major depression: a randomized controlled trial

**DOI:** 10.1186/s12888-018-1788-6

**Published:** 2018-06-22

**Authors:** Fujika Katsuki, Hiroshi Takeuchi, Takahiko Inagaki, Tohru Maeda, Yosuke Kubota, Nao Shiraishi, Hideaki Tabuse, Tadashi Kato, Atsurou Yamada, Norio Watanabe, Tatsuo Akechi, Toshiaki A. Furukawa

**Affiliations:** 1 Department of Psychiatric and Mental Health Nursing, Nagoya City University School of Nursing, Kawasumi, Mizuho-cho, Mizuho-ku, Nagoya, Japan; 2 Department of Psychiatry, Japanese Red Cross Nagoya Daini Hospital, Myokencho 2-9, Syowa-ku, Nagoya, Japan; 3 Department of Psychiatry, Shiga University of Medical Science, Seta Tsukinowa cho, Otsu, Shiga Japan; 4 School of Pharmacy, Kinjo Gakuin University, 2-1723 Omori Moriyama-ku, Nagoya, Japan; 5 Department of Psychiatry and Cognitive-Behavioral Medicine, Nagoya City University Graduate School of Medical Sciences, 1 Kawasumi, Mizuho-cho, Mizuho-ku, Nagoya, Japan; 6 Holy Cross Hospital, Kujiri 2431-160, Izumi-cho, Toki, Gifu, Japan; 7 Aratama Kokoro Clinic, Suyama-cho 1-49, Mizuho-ku, Nagoya, Japan; 8 Department of Health Promotion and Human Behavior, Kyoto University Graduate School of Medicine/School of Public Health, Yoshida Konoe-cho, Sakyo-ku, Kyoto, Japan

**Keywords:** Major depressive disorder, Family psychoeducation, Randomized controlled trial

## Abstract

**Background:**

Major depressive disorder (MDD) is a common and often chronic problem. Patients with chronic MDD often have negative impacts on the health of their families. Family psychoeducation is recognized as part of the optimal treatment for patients with psychotic disorder, and has been shown to reduce the rate of relapse in individuals with schizophrenia and to reduce the burden on their caregivers. Thus, we predict that family psychoeducation has the potential to reduce the burden on the caregivers of patients with chronic MDD. In the present study, we aimed to investigate the effects of brief multifamily psychoeducation (BMP) on the mental health status of family members of patients with chronic MDD.

**Methods:**

We conducted a clinical trial consisting of 49 chronic MDD patients and their families. Each family was randomly assigned to either the BMP intervention group or the control group. The intervention group received four BMP sessions, once every two weeks for eight weeks. The control group received one counseling session administered by a nurse. All patients received standard treatment administered by physicians. The primary outcome measurement was the Kessler Screening Scale for Psychological Distress (K6) score of family members at 16- weeks after the first BMP session. Secondary outcomes were depressive symptoms of both family members and patients at multiple time points, as well as family functioning as evaluated by the patients. Intention-to-treat analyses were conducted.

**Results:**

There was no statistically significant effect of BMP on K6 scores at 16- weeks (mean difference 1.17, 95% confidence interval: − 0.63 to 2.98, *P* = 0.19). Exploratory analyses revealed that BMP reduced depressive symptoms in family members at 8- weeks (difference = − 3.37, 95%CI -6.32 to − 0.43, *P* = 0.02) and improved family functioning at multiple time points (Role; 8 W, difference = − 0.13, 95%CI -0.26 to − 0.00, *P* = 0.04, Affective Responsiveness; 8 W, difference = − 0.24, 95%CI -0.43 to − 0.05, *P* = 0.01, 32 W, difference = − 0.22, 95%CI -0.41 to − 0.03, P = 0.02, Behavior Control; 16 W, difference = − 0.17, 95%CI -0.34 to − 0.00, P = 0.04).

**Conclusions:**

Four BMP sessions did not significantly reduce the psychological distress of family members of patients with chronic MDD.

**Trial registration:**

Clinical Trials. gov NCT01734291, retrospectively registered (Registration date: November 21, 2012).

## Background

Long-term course for major depressive disorder (MDD) is poor even after adequate treatment. Twenty percent of patients had chronic depressive symptoms for more than one year after starting treatment in Japanese cohort study [1]. Even once recovered, the probability of remaining symptom-free was 57% at 1 year, 47% at 2 years, and 35% at 5 years [2]. This illustrates a real danger of becoming chronically depressed after initial MDD diagnosis. For those with chronic MDD, it can mean a great deal of suffering for their families, including a higher divorce rate between patients and spouse [3, 4] and severe financial strain [5] . Close family members of patients with chronic depression are therefore more likely to develop subthreshold depression, which is a strong risk factor for MDD [6]. In fact, Benazon and Coyne [7] showed that depressed patient mood was a significant predictor of depressed spouse mood. In our previous study [8], using the Kessler Screening Scale for Psychological Distress (K6), which measures general psychological distress, including depression and anxiety, we demonstrated that families of patients with MDD had significantly worse mental health status than the general population of Japan (K6 scores 8.6 ± 5.4 versus 3.6 ± 3.9) [9]. It is therefore of great interest to reduce psychological burden among the family members of patients with chronic MDD. However, there is no commonly accepted strategy that is proven effective for this purpose.

Family psychoeducation is recognized as an important part of optimal treatment, along with traditional medication and counseling, for patients with a psychotic disorder [10, 11]. Family psychoeducation has been shown to reduce the rate of relapse and hospitalization in individuals with schizophrenia and to reduce the burden on their caregivers [12, 13]. Patients with MDD who were in partial or full remission whose families underwent multifamily psychoeducation, had a significantly lower rate of relapse than control patients [14]. Lemmens et al. [15] found that patients with MDD who were treated with multifamily psychoeducation in addition to traditional treatment had higher response rates than patients receiving traditional treatment alone. In a study of adolescents with MDD, patients who received family psychoeducation showed significant improvements in social functioning and adolescent-parent relationships over standard treatment [16]. Taken together this suggests that family psychoeducation has the potential to reduce the burden on caregivers and to even improve patient outcomes. No study, however, has yet investigated the effect of this treatment on mental health in the family members of patients with MDD. Here, we performed a randomized control trial (RCT) to examine the effectiveness of brief multifamily psychoeducation (BMP) on improving the mental health status of family members of patients with chronic MDD. We hypothesized that, compared to families receiving one regular counseling session by a nurse, families receiving BMP would show greater improvements in mental health status, or maintain good mental health status at 8 weeks after the four BMP sessions.

## Methods

### Participants

We recruited patients with MDD of more than one year’s duration and their primary family members, i.e., the fathers, mothers, husbands, wives, daughters, and sons of patients. Inclusion criteria were: the patient met the criteria for MDD (currently in full episode or in partial remission) according to DSM-IV based on the consensus rating by trained psychiatrists (who had more than 5 years of experience as a physician and more than 2 years of experience as a psychiatrist); the patient was receiving antidepressant therapy at the time of entry into the study; the patient had the first episode of MDD more than one year prior to study recruitment; the patient and his/her family member(s) were aged between 18 and 85 years; the patient lived with his/her family at the time of entry in this study and was expected to live with his/her family during the study period. Patients who were undergoing electroconvulsive therapy during the study period and patients who were at serious risk of suicide were excluded from this study.

Participants were recruited at the Department of Psychiatry, Nagoya City University Hospital, Japan, the Department of Psychiatry, Shiga University of Medical Sciences, Holy Cross Hospital, Yagoto Hospital and Aratama Kokoro Clinic between October 1, 2012 and January 20, 2016, and were followed up until October 9, 2016. We asked doctors in these hospitals to introduce our research group to inpatients and outpatients who were undergoing treatment for their MDD, and we recruited the patients and their family members who had shown interest to participate in this study.

### Procedure

This study was approved by the Ethics Review Committee of Nagoya City University Graduate School of Medicine, Japan (Ref: No.679) and the participating clinics and hospitals, and was conducted in accordance with the principles stated in the Helsinki Declaration. The study is registered at ClinicalTrials.gov under number NCT01734291 (November 21, 2012). The protocol for this trial was reported previously [17].

We provided patients with an ID number and then patients and their families were given an explanation of the purpose and the procedure of the study using leaflets. After reading the leaflets, both patients and their families were provided with the complete study description before being asked to provide written consent to participate in the study. Up to three members per family were allowed to receive the family psychoeducation or standard therapy, although only one family member in each family underwent the evaluation in the present study. We asked the family members of each family to decide which family member would be evaluated and all assessments were administered with that family member.

After providing consent and completing the baseline assessment, the patient-family units were randomized. Participants were randomly allocated, with equal probability (1:1), of being assigned to either the intervention group (family members received four BMP sessions) or the control group (family members received one counseling session administered by a nurse). In either case patients received standard treatment. An independent statistician generated the random allocation sequences, stratified by the family member’s severity of mental distress (K6 score of 5 or more, or less than 5), using minimization on a computer [18]. Allocation sequences were kept by the statistician centrally, and the allocation was facsimiled to us after the research assistant ascertained the eligibility and the written informed consent. The randomization schedule was not available to anyone except the statistician. The patients in both the treatment and control groups received standard outpatient or inpatient treatment administered by physicians. In the MDD patients, outpatient treatment consisted of evaluation of psychiatric symptoms, antidepressant pharmacotherapy and supportive psychotherapy on an every-other-week or every-four-week basis. Inpatient treatment consisted of sufficient rest for the patient, evaluation of psychiatric symptoms, antidepressant pharmacotherapy and supportive psychotherapy. Some case management was provided to all participants.

### Intervention

#### Brief multifamily psychoeducation (BMP)

We developed the ‘brief multifamily psychoeducation’ program based on the McFarlane Model [19], the Evidence-Based Practices Toolkit for Family Psycho-Education (EBP Toolkit) [20], and the standard model of the Japanese Network of Psychoeducation and Family Support Program (JNPF) [21]. Our family intervention program is similar to the McFarlane Model in that our program is administered to a group of families and problem-solving is used. In the McFarlane Model, patients with MDD are included in the multifamily group, but in the current study, we did not include patients with MDD in the family groups because we feared that patients would feel guilty towards the family. Our program was therefore shorter than that by McFarlane (4 sessions vs 7 sessions including one screening session).

The staff members who participated in the BMP consisted of one or two psychiatrists, one or two nurses, one pharmacologist and one social worker or psychologist. The BMP program was divided into four sessions; we chose four sessions as the minimum number of sessions based on the study by Shimazu et al. [14] to reduce families’ burden, with each session consisting of the families of approximately four patients. We have examined the feasibility of the BMP program in a pilot one-arm study with 32 participants and found statistically significant improvements in families’ mental health status [8]. Each BMP session consisted of a lecture, followed by supportive group therapy focusing on problem-solving skills for approximately 90 min. At the first BMP session, we gave the participants information on the causes and symptoms of major depression; at the second session, we provided information on the various drugs that are used to treat MDD; at the third session, we provided information on community resources that provide assistance to families of patients with MDD; and at the fourth session, we provided guidelines for families caring for MDD patients. Each session was conducted by three or four BMP team members. The group leader and co-leaders encouraged the family members to give a narrative of their subjective experience in taking care of their family member with MDD. In accordance with the standard model of the JNPF, our supportive group therapy consisted of the following four steps: (i) families socializing with other families, (ii) group members were asked to present problems or goals, (iii) for each problem or goal, group members discuss and suggest possible solutions, and (iv) the family member who presented the problem chooses the solution that best fits the situation. Each BMP session of lecture and supportive group therapy lasted approximately 2 h. The groups met once every two weeks. The family members were given a booklet developed by our department and a videotape produced by the Department of Neuropsychiatry at Kochi Medical School [22], which included an interview of the experience of a patient with MDD and an explanation of the molecular cause of MDD using computer graphics of synapses and neurotransmitters.

#### Control group

Family treatment in the control group consisted of one counseling session administered by one of four nurses who each had more than 10 years’ experience as a psychiatric nurse. We selected this counseling treatment as the treatment for the control group, because active listening to relatives’ suffering and giving information on recuperation by nurses are within the treatment as usual. The number of session was limited to one, as would be practiced within the framework of treatment as usual in Japan. In this counseling session, the nurse provided information only when each family requested such. Often, requested information was about how to best communicate, avoid relapses, and drug-related questions. The one session of counseling by a nurse lasted 45 min.

#### Therapist training/supervision and Fidelity control

The first, third and sixth authors of this study were each trained and certified as family psychoeducation instructors by the JNPF [21]. All staff, except the one pharmacologist, received more than eleven hours of intensive training using the treatment manual of the JNPF. To ensure the fidelity of the trial, we audio-recorded all multifamily psychoeducation sessions and control counseling sessions, and we evaluated the quality of treatment in the randomly selected 25 and 20%, respectively, of the sessions. Independent researchers who were not in charge of that session evaluated treatment quality using checklists. They had participated in the intensive training of the JNPF as an instructor prior to participation in the current study. The checklists using fidelity check in this study were created by the National Project Team of Japan and modified by our group [23].

### Outcome measures

The assessments were conducted at pretreatment, and at 8, 16, and 32 weeks since the trial initiation (i.e. at post-treatment, 8 weeks post-treatment and 24 weeks post-treatment).

### Primary outcome measure of family members

The primary outcome was the mental health status of family members at 16 weeks post-randomization. To measure this, we used the K6 questionnaire, a six-item self-report questionnaire that was developed to screen for *DSM-IV* defined depression and anxiety disorders within 30 days prior to its administration. K6 can also be used to quantify nonspecific psychological distress [24]. Each of the six questions in the K6 questionnaire is rated from 0 = (“none of the time”) to 4 = (“all of the time”), and the total score therefore ranges from 0 (no psychological distress) to 24 (severe psychological distress). Two independent validation studies of K6 [24, 25], and the Japanese version of the K6 questionnaire showed excellent validity [26]. Cronbach’s α coefficient of reliability in this study was 0.82.

### Secondary outcomes of family members

#### The Japanese version of the Zarit burden interview short version (J-ZBI_8)

The Zarit Burden Interview (ZBI) which was originally developed to assess the burden of relatives of impaired elderly people, is widely used to assess the burden of caregivers [27]. Arai et al. [28] developed the Japanese version of the ZBI, the J-ZBI, which consists of 22items, and also the eight-item short version of the J-ZBI (J-ZBI_8) [29, 30]. The items in the J-ZBI_8 are rated on a 5-point Likert scale (0 = never to 4 = very often) and the scores on the J-ZBI_8 range from 0 to 32, with higher scores indicating greater burden. Arai et al. [30] reported that Cronbach’s α of the J-ZBI_8 was 0.89, and the Pearson’s correlation coefficient between scores on the J-ZBI and J-ZBI_8 was 0.93. Cronbach’s α coefficient of reliability in the present study was 0.84.

#### The Japanese version of the family attitude scale (FAS)

The FAS, developed by Kavanagh, et al. [31], is a 30-item self-report inventory that measures families’ Expressed Emotion (EE). The total score on the FAS ranges from 0 to 120, with higher scores indicating higher levels of burden or criticism [31]. Higher FAS rating was significantly correlated with higher levels of criticism (*r* = 0.44), hostility (*r* = 0.41) and emotional overinvolvement (EOI) (*r* = 0.27) in the Camberwell Family Interview (CFI) [32]. Fujita et al. [33] developed the Japanese version of the FAS, which showed excellent validity. The relative sensitivity and specificity of the EE assessment on the FAS compared with the criticism component of the CFI were 100 and 88.5%, respectively, in a study on relatives of patients with schizophrenia in Japan [33]. Cronbach’s α coefficient of reliability in the present study was 0.79.

#### Beck depression inventory: BDI-II

BDI-II is a 21-item self-report instrument that assesses the presence and severity of symptoms of depression [34]. Each item is rated on a 4-point scale ranging from 0 to 3, with a high score representing severe symptoms of depression. The BDI-II is a reliable, internally consistent, and valid scale for assessing depression [34–36]. The reliability and validity of the Japanese version are excellent [37]. Cronbach’s α coefficient of reliability in this study was 0.80.

### Secondary outcomes of patients

#### Beck depression inventory: BDI-II

The BDI-II was selected as an outcome measure to evaluate the severity of patients’ depressive symptoms. Cronbach’s α coefficient of reliability in this study was 0.93.

#### The MOS 36-item short form health survey (SF-36) version 2

The SF-36 is a 36-item self-report questionnaire that assesses the general quality of life across eight domains. The eight domains are Physical Functioning (PF), Role Physical (RP), Bodily Pain (BP), Social Functioning (SF), Role Emotional (RE), General Health Perceptions (GH), Vitality (VT), and Mental Health (MH). It also provides two summary scores, the Physical Component Summary (PCS) and the Mental Component Summary (MCS). The PCS is associated with PF, RP, BP, GH and VT. The MCS is associated with MH, RE, SF, VT and GH. The score of each of the 8 domains ranges from 0 to 100, with higher scores indicating higher quality of life. The Japanese version of the SF-36 has shown good validity in the general population of Japan [38, 39]. Cronbach’s α coefficient of reliability in this study was 0.92 for PF, 0.93 for RP, 0.91 for BP, 0.64 for SF, 0.89 for RE, 0.79 for GH, 0.74 for VT, and 0.77 for MH.

#### The Japanese version of the family assessment device (FAD)

The FAD is a self-report questionnaire developed by Epstein et al. [40] that assesses the six dimensions of the McMaster Model of Family Functioning as well as the family’s overall level of functioning. This questionnaire has 60 items and each item is scored from 1 (strongly disagree) to 4 (strongly agree) based on the Likert scale. The FAD consists of the following seven subscales: Problem Solving, Communication, Roles, Affective Responsiveness, Affective Involvement, Behavior Control, and General Functioning. Saeki et al. [41] developed the Japanese version of the FAD, which showed good validity. The higher the score of each subscale, the lower the family function of that area. In this study, we administered the FAD to the MDD patients only because we thought that the FAS that was administered to family members would provide information similar to the FAD. Cronbach’s α coefficient of reliability in this study was 0.86 for Problem Solving, 0.69 for Communication, 0.78 for Roles, 0.76 for Affective Responsiveness, 0.58 for Affective Involvement, 0.68 for Behavior Control, and 0.84 for General Functioning.

### Sample size and statistical power

To determine the appropriate sample size for this study, we performed a power analysis on K6 scores in our previous study (mean change in K6 scores was 4.9 in 32 participants) [8]. The change in K6 scores pretreatment to posttreatment (16 weeks after the randomization) was 4.5 ± 2.5 (mean ± SD) in the family psychoeducation group and 2 ± 2.5 in the control group. The sample size needed to detect a significant difference at a level of *P* = 0.05 (2-sided) with a power of 0.9 was 23 participants in each group. We predicted a 20% dropout rate, and we decided to recruit 30 participants for each group. However, in the course of the trial it was found that the dropout rate at the time of the primary outcome assessment was extremely low (dropout rate, 3%). We therefore adjusted the required sample size based on this observed dropout rate and decided to set a new target number of participants at 48 in total, in July 2015.

### Statistical analysis

We used SAS PROC MIXED (Version 9.4, SAS Institute) to conduct the maximum likelihood linear mixed model for repeated measures (MMRM) analyses of the primary and secondary outcomes to account for missing data. The model included fixed effects for treatment, visit, and treatment*visit, adjusted for the covariates and the respective scale’s baseline values, and random effects for participants. Descriptive data analysis was conducted by calculating mean scores and standard deviation. All analyses were based on the intent-to-treat model. An alpha of 0.05 was set to test against the null hypothesis. We did not adjust α-levels for secondary outcomes, because these analyses were exploratory. We conducted secondary analyses for exploratory purposes and did not adjust for multiple testing. No statistical tests were planned to detect a difference at baseline between the two arms because we aimed to avoid multiple tests and the decision to adjust for baseline data in RCTs should not be determined by whether baseline differences are statistically significant [42]. Statistical analysis was performed by a blinded statistician.

## Results

### Participants and baseline characteristics

**Fig. 1:**
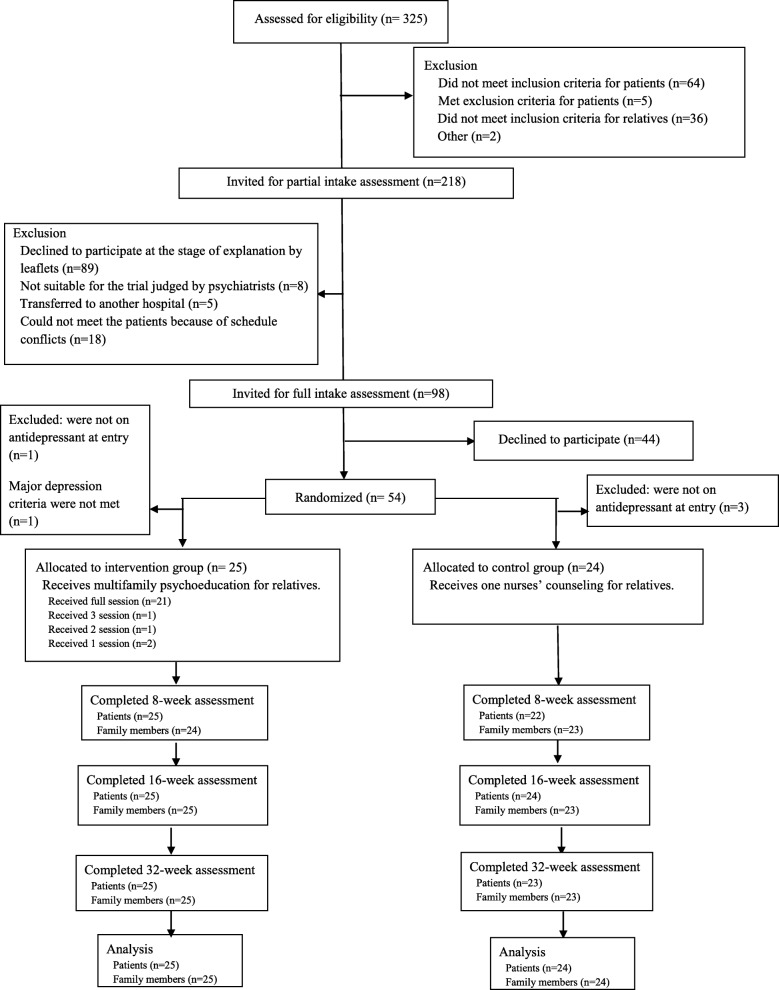
Participant Flow Diagram

Of the 325 patients that were screened, 54 patients and their families were randomized for the study. After randomization, 2 were excluded from the intervention group (one was not on antidepressants at entry and one did not meet major depression criteria) and 3 were excluded from the control group (they were not on antidepressants at entry). Members of the research team mistakenly enrolled 4 patients who had used antidepressants in the past but were not using antidepressants at the time of study entry. One patient was introduced and entered in the study by a psychiatrist who was not a research team member. We checked the medical records of this patient later, and we could not confirm his MDD diagnosis. Post-hoc exclusion of patients for reasons prior to randomization (i.e. patients mistakenly randomized) is a permitted practice in the analysis and interpretation of randomized controlled trials [43]. The 49 patients and their families were then split into the BMP group (25 patients and families) and standard treatment group (24 patients and families). All patients and family data are available for analysis (Fig. 1). Table 1 summarized the socio demographic and clinical parameters at baseline of the 49 patients and their family members. Up to three family members per family were permitted to receive the BMP therapy or standard therapy; however, there was only one family in which more than one family member participated in the trail.

**Table 1:** Characteristics of patients and family members at baseline

Characteristics	intervention group	control group	all participants
	*n* = 25	*n* = 24	*n* = 49
Family members			
Age, mean (SD), y	55.4 (14.5)	57.2 (13.2)	56.3 (13.8)
Sex, n (%)			
Female	18 (72.0)	15 (62.5)	33 (67.3)
Male	7 (28.0)	9 (37.5)	16 (32.7)
Family relationship, n (%)			
father	1 (4.0)	2 (8.3)	3 (6.1)
mother	8 (32.0)	9 (37.5)	17 (34.6)
husband	5 (20.0)	6 (25.0)	11 (22.4)
wife	10 (40.0)	5 (20.8)	15 (30.6)
daughter	0	1 (4.2)	1 (2.0)
son	1 (4.0)	1 (4.2)	2 (4.0)
K6, mean (SD)	5.2 (3.3)	5.6 (4.4)	5.4 (3.9)
BDI-II, mean (SD)	11.4 (5.8)	11.6 (7.2)	11.5 (6.5)
J-ZBI_8, mean (SD)	8.3 (6.1)	8.6 (5.6)	8.4 (5.8)
FAS, mean (SD)	43.4 (19.9)	41.2 (20.2)	42.3 (19.8)
Patient			
Age, mean (SD), y	43.5 (17.4)	43.9 (18.2)	43.7 (17.6)
Sex, n (%)			
Female	11 (44.0)	13 (54.2)	24 (49.0)
Male	14 (56.0)	11 (45.8)	25 (51.0)
Duration of treatment for index episode, mean (SD), y	7.0 (6.4)	9.2 (8.4)	8.1 (7.5)
Number of admissions, mean (SD)	0.8 (1.1)	1.7 (3.4)	1.2 (2.5)
Experience of ECT treatment, n (%)	4 (16.0)	3 (12.5)	7 (14.3)
Antidepressant usage, mean (SD), DDD	1.7 (0.7)	1.6 (0.8)	1.7 (0.7)
Hypnotics usage, mean (SD), DDD	0.7 (1.0)	0.5 (0.9)	0.6 (0.9)
Antianxiety usage, mean (SD), DDD	0.1 (0.2)	0.2 (0.5)	0.2 (0.4)
Antipsychotics, n (%)	7 (28.0)	5 (20.8)	12 (24.5)
Lithium, n (%)	5 (20.0)	6 (25.0)	11 (22.4)
BDI-II, mean (SD)	22.2 (10.4)	25.7 (15.1)	24.0 (12.9)
FAD, mean (SD)			
PS (Problem Solving)	2.4 (0.6)	2.5 (0.6)	2.5 (0.6)
CM (Communication)	2.2 (0.3)	2.3 (0.5)	2.2 (0.4)
RL (Roles)	2.0 (0.4)	2.1 (0.5)	2.0 (0.4)
AR (Affective Responsiveness)	2.2 (0.5)	2.1 (0.6)	2.2 (0.5)
AI (Affective Involvement)	2.1 (0.4)	2.1 (0.3)	2.1 (0.4)
BC (Behavior Control)	2.2 (0.3)	2.2 (0.4)	2.2 (0.4)
GF (General functioning)	2.1 (0.5)	2.2 (0.6)	2.1 (0.5)
SF-36, mean (SD)			
PCS (Physical component summary)	48.6 (15.4)	48.6 (15.4)	46.8 (15.4)
MCS (Mental component summary)	42.2 (8.9)	42.2 (9.8)	42.2 (9.2)

### Study integrity

Of the 9 randomly selected sessions in the intervention group checked for adherence, 87.2% of the quality checkpoints were fulfilled by the therapist. Of the 4 randomly selected sessions in the control group checked for adherence, 100% of the quality checkpoints were fulfilled by the nurse. The average session length in the control group was 51 ± 10 (mean ± SD) minutes.

### Primary outcome

Table 2 shows the family-member group Latest Square (LS) means and their 95% confidence intervals for the K6 score at 8, 16, and 32 weeks from the start of treatment, adjusted for stratification variables, family member-patient relationship, family member age, K6 baseline scores, and random effects for family members in a maximum likelihood mixed effects model.

**Table 2:** Estimated mean outcome scores at 8, 16, 32 weeks

			intervention guroup	control group	Defference(95% CI)	*P* Value
Family members						
Questionnaire, LS mean (95% CI)						
K6		8 W	4.27 (2.78 to 5.77)	5.33 (3.96 to 6.71)	−1.05 (−2.68 to 0.56)	0.19
		16 W	5.65 (4.06 to 7.23)	4.47 (2.97 to 5.98)	1.17 (−0.63 to 2.98)	0.19
		32 W	4.82 (3.23 to 6.41)	4.34 (2.85 to 5.83)	0.48 (−1.32 to 2.29)	0.59
BDI-II		8 W	7.36 (3.98 to 10.75)	10.74 (7.61 to 13.86)	−3.37 (−6.32 to − 0.43)	0.02
		16 W	8.49 (4.54 to 12.45)	10.34 (6.72 to 13.96)	−1.84 (−6.03 to 2.34)	0.37
		32 W	9.36 (5.19 to 13.53)	8.41 (4.53 to 12.28)	0.94 (−3.66 to 5.55)	0.67
J-ZBI_8		8 W	7.35 (5.39 to 9.31)	6.14 (4.33 to 7.95)	1.21 (−0.94 to 3.36)	0.26
		16 W	7.06 (4.88 to 9.25)	5.21 (3.10 to 7.32)	1.85 (−0.73 to 4.44)	0.15
		32 W	5.93 (3.57 to 8.30)	4.04 (1.77 to 6.31)	1.89 (−0.97 to 4.76)	0.18
FAS		8 W	41.19 (30.22 to 52.17)	41.25 (31.08 to 51.42)	−0.05 (−10.84 to 10.72)	0.99
		16 W	41.33 (29.60 to 53.07)	37.18 (26.31 to 48.05)	4.15 (−8.26 to 16.58)	0.50
		32 W	39.86 (28.49 to 51.22)	32.36 (22.04 to 42.68)	7.49 (−4.18 to 19.17)	0.20
patient						
Questionnaire, LS mean (95%CI)						
BDI-II		8 W	21.61 (17.50 to 25.72)	23.51 (19.32 to 27.70)	−1.91(−7.85 to 4.05)	0.52
		16 W	19.98 (15.86 to 24.09)	21.67 (17.62 to 25.72)	−1.69 (−7.55 to 4.16)	0.56
		32 W	19.14 (15.19 to 23.09)	20.34 (16.32 to 24.36)	−1.19 (−6.89 to 4.50)	0.67
FAD	PS	8 W	2.33 (2.19 to 2.46)	2.46 (2.33 to 2.60)	−0.13(−0.33 to 0.05)	0.16
		16 W	2.33 (2.17 to 2.49)	2.41 (2.24 to 2.57)	−0.07 (− 0.3 to 0.15)	0.52
		32 W	2.15 (2.00 to 2.31)	2.33 (2.16 to 2.49)	−0.17 (− 0.40 to 0.05)	0.13
	CM	8 W	2.31 (2.19 to 2.43)	2.33 (2.20 to 2.45)	−0.01(− 0.19 to 0.15)	0.82
		16 W	2.29 (2.17 to 2.41)	2.33 (2.21 to 2.45)	−0.04 (− 0.21 to 0.13)	0.63
		32 W	2.25 (2.11 to 2.39)	2.22 (2.08 to 2.37)	0.02 (−0.17 to 0.22)	0.79
	RL	8 W	2.01 (1.92 to 2.10)	2.14 (2.05 to 2.23)	−0.13(− 0.26 to − 0.00)	0.04
		16 W	1.99 (1.87 to 2.11)	2.09 (1.97 to 2.21)	−0.09 (− 0.27 to 0.07)	0.25
		32 W	2.00 (1.90 to 2.10)	2.07 (1.97 to 2.18)	−0.07 (− 0.22 to 0.07)	0.33
	AR	8 W	2.17 (2.04 to 2.30)	2.42 (2.28 to 2.55)	−0.24(− 0.43 to − 0.05)	0.01
		16 W	2.13 (1.98 to 2.28)	2.30 (2.15 to 2.45)	−0.16 (− 0.38 to 0.04)	0.12
		32 W	2.12 (1.99 to 2.25)	2.34 (2.21 to 2.48)	−0.22 (− 0.41 to − 0.03)	0.02
	AI	8 W	2.22 (2.06 to 2.33)	2.31 (2.18 to 2.45)	−0.11(− 0.30 to 0.07)	0.22
		16 W	2.14 (2.00 to 2.27)	2.21 (2.07 to 2.35)	−0.07 (− 0.27 to 0.12)	0.43
		32 W	2.11 (1.98 to 2.24)	2.21 (2.08 to 2.34)	−0.09 (− 0.28 to 0.08)	0.28
	BC	8 W	2.09 (1.97 to 2.20)	2.25 (2.13 to 2.36)	−0.15(− 0.32 to 0.00)	0.05
		16 W	2.10 (1.98 to 2.22)	2.27 (2.15 to 2.39)	−0.17 (− 0.34 to − 0.00)	0.04
		32 W	2.08 (1.96 to 2.20)	2.23 (2.11 to 2.36)	−0.15 (− 0.32 to 0.01)	0.07
	GF	8 W	2.10 (1.99 to 2.20)	2.18 (2.07 to 2.29)	−0.08(− 0.24 to 0.07)	0.28
		16 W	2.09 (1.94 to 2.25)	2.16 (2.00 to 2.31)	−0.06 (− 0.28 to 0.15)	0.56
		32 W	1.99 (1.84 to 2.13)	2.05 (1.89 to 2.20)	−0.05 (− 0.27 to 0.15)	0.58
SF-36	PCS	8 W	45.48 (41.64 to 49.32)	46.79 (42.81 to 50.77)	−1.30(−6.87 to 4.26)	0.63
		16 W	43.35 (39.97 to 46.72)	44.14 (40.71 to 47.57)	−0.79 (−5.66 to 4.06)	0.74
		32 W	46.28 (43.48 to 49.08)	44.91 (41.99 to 47.83)	1.36 (−2.71 to 5.44)	0.50
	MCS	8 W	43.01 (39.61 to 46.42)	40.36 (36.84 to 43.88)	2.65(−2.29 to 7.60)	0.28
		16 W	46.25 (43.37 to 49.13)	44.58 (41.66 to 47.51)	1.66 (− 2.51 to 5.83)	0.42
		32 W	45.54 (41.27 to 49.81)	45.20 (40.76 to 49.65)	0.33 (−5.85 to 6.52)	0.91
Medications, LS mean (95%CI), DDD						
Antidepressants		8 W	1.66 (1.51 to 1.81)	1.70 (1.55 to 1.85)	−0.03 (−0.24 to 0.17)	0.74
		16 W	1.69 (1.50 to 1.87)	1.65 (1.46 to 1.84)	0.04 (−0.22 to 0.30)	0.76
		32 W	1.38 (1.13 to 1.64)	1.47 (1.21 to 1.73)	−0.08 (− 0.45 to 0.27)	0.63
Antianxiety		8 W	0.19 (0.12 to 0.26)	0.12 (0.04 to 0.19)	0.06 (−0.03 to 0.17)	0.18
		16 W	0.19 (0.12 to 0.26)	0.12 (0.04 to 0.19)	0.06 (−0.03 to 0.17)	0.18
		32 W	0.15 (0.08 to 0.22)	0.15 (0.08 to 0.22)	0.00 (−0.10 to 0.10)	0.98
Hypnotics		8 W	0.51 (0.31 to 0.72)	0.70 (0.49 to 0.91)	−0.18 (− 0.48 to 0.11)	0.21
		16 W	0.51 (0.29 to 0.73)	0.64 (0.41 to 0.87)	−0.12 (− 0.45 to 0.19)	0.43
		32 W	0.39 (0.16 to 0.63)	0.68 (0.44 to 0.92)	−0.28 (− 0.63 to 0.05)	0.09

The adjusted model includes fixed effects fortreatment, visit, and treatment*visit, adjusted for the family member’s relationship with the patient, the family member’s age and the scale’s baseline score and random effects for family members. The adjusted model includes fixed effects for treatment, visit, and treatment*visit, adjusted for the patient’s age, sex, duration of illness, number of hospitalizations, patient status (outpatient vs inpatient), antidepressant use and the scale’s baseline score and random effects for patients

*Abbreviations*: *LS means* latest square means, *8 W* 8 weeks, *16 W* 16 weeks, *32 W* 32 weeks, *BDI-II* Beck depression inventory, *J-ZBI_8* the Japanese version of the Zarit burden interview short version, *FAS* family attitude scale, *FAD* family assessment device, *PS* problem solving, *CM* communication, *RL* roles, *AR* affective responsiveness, *AI* affective involvement, *BC* behavior control, *GF* general functioning, *PCS* physical component summary, *MCS* mental component summary, *DDD* defined daily dose

With respect to the primary outcome of the K6 score at 16 weeks for family members, there was no significant difference between the intervention and control groups (difference = 1.17, 95%CI -0.63 to 2.98, *P* = 0.19).

### Secondary outcome of family members

With respect to BDI-II scores of family members at 8 weeks, the intervention group was lower than the control group (difference = -3.37, 95%CI -6.32 to − 0.43, *P* = 0.02). With respect to the J-ZBI_8 and FAS score of family members, there were not any differences (Table 2).

### Secondary outcome of patients

Table 2 also shows the group LS means and their 95% confidence intervals for the BDI-II, FAD, SF-36 and the defined daily dose of some medications at 8, 16, and 32 weeks from the start of treatment, adjusted for the stratification variables, the patient’s age, sex, duration of illness, number of hospitalizations, patient status (outpatient vs inpatient), antidepressant use and the scale’s baseline score and random effects for patients in a maximum likelihood mixed effects model.

In some subscales of FAD evaluated by patients, the score of the intervention group was lower than the control group at several points (Role; 8 W, difference = -0.13, 95%CI -0.26 to − 0.00, *P* = 0.04, Affective Responsiveness; 8 W, difference = -0.24, 95%CI -0.43 to − 0.05, *P* = 0.01, 32 W, difference = -0.22, 95%CI -0.41 to − 0.03, P = 0.02, Behavior Control; 16 W, difference = -0.17, 95%CI -0.34 to − 0.00, P = 0.04). With respect to the BDI-II and SF-36 score of patients and defined daily dose of medication, there were not any differences (Table 2).

## Discussion

The present study examined the effectiveness of BMP in improving the mental health status of families of patients with chronic MDD. There was no significant benefit of BMP intervention on the primary outcome measure, i.e. mental health status of chronic MDD family members at 16 weeks. In the exploratory examinations, some family functioning, measured by the patient-evaluated FAD (Roles, Affective Responsiveness, Behavior Control) in the intervention group was better than those in the control group at several evaluation points. We found no effect of BMP on depressive symptoms of chronic MDD patients.

With regard to family members, we observed no statistically significant benefit of BMP intervention. This failure to differentiate was probably due to the participation of relatively mentally healthy individuals with low baseline scores (the K6 score in the general population has been found to be 3.6 ± 3.9 [9] and a BDI-II score of 13 points or less indicates minimal depression [44]). It is possible that chronic MDD family members with the highest distress may not have participated in this research. In the intervention group, once the K6 and BDI-II scores of the family members improved, the effect seemed to peak at 8 weeks, then decreased afterward. This suggests that family members of chronic MDD patients may not learn enough about how to deal with the daily stress of having a loved one suffering from MDD in only 4 sessions and the termination of the program may have even increased their anxiety and depression. Continuous group sessions may be needed to improve the mental health of family members of patients with chronic MDD.

We saw an improvement trend in family function (Roles, Affective Responsiveness, and Behavior Control) recognized by the patients as a result of BMP. Previous work supports these results where, compared to controls, adolescent MDD patients receiving family psychoeducation had improved family function as measured by patient-evaluated FAD (Communications, Affective Involvement) [16]. Their results suggest that family psychoeducation creates a positive change in family function that is recognized by MDD patients. It is this mechanism that may be behind the relapse prevention effect of MDD family psychoeducation [14].

In the present study, BMP intervention did not have a significant effect on the EE of family members as measured by the FAS at all assessment points. With regard to schizophrenia, a meta-analysis of studies on the families of schizophrenia patients showed that the family’s EE is a good predictor of relapse in schizophrenia [45]. On the other hand, with regard to MDD, there have been fewer studies on the relationship between the family’s EE and the course of MDD in patients. Three studies [46–48] reported that high EE predicted a worse consequence in MDD patients, while one study [49] reported that there was no clear association between the EE of a spouse and recurrence of depression in the patient. A previous study supports these results where neither EE status nor FAS scores at 9-months follow-up differed significantly between the family psychoeducation intervention group and control group [14] . Therefore, with regard to MDD, it is not clear whether a family’s high EE predicts recurrence in the patient, and whether the family psychoeducation reduces the EE status of family members.

To date, there have been five RCTs on family psychoeducation of MDD [14–16, 50, 51], but only three of which were strict trials where the primary outcome was clearly stated [14–16]. In the present study, we found no effect of BMP on depressive symptoms of patients (BDI-II baseline mean score was 24). Similarly, the Sanford study [16] reported that family psychoeducation had no effect on depressive symptoms in MDD patient. The Lemmens study [15] reported that multifamily group therapy intervention had no effect on depressive symptoms measured by BDI-II at 3- or 15-months or on remission rate at 3- or 15-months (BDI-II baseline mean score was 27). But this intervention led to higher treatment response rates at 15-months in MDD patients compared to controls [15]. In addition, the patients who had achieved full or partial remission from an acute depressive episode (BDI-II baseline mean score was 12) had a significantly lower relapse rate during a 9-month follow-up period compared to controls [14]. Further, the aforementioned studies were relatively similar in design. Our study and the Shimazu study [14] were almost identical, consisting of four sessions of family psychoeducation and group family treatment. The Lemmens study [15] had seven sessions of multifamily psychoeducation, and the Sanford study [16] had twelve sessions of single family psychoeducation. Therefore, despite similar psychoeducation designs, the few available RCTs comparing family psychoeducation have produced heterogeneous results. Further RCTs of family psychoeducation for MDD are needed to clarify this.

One limitation of this study was that we overestimated the effect size. That is, we calculated sample size based on a power analysis of K6 scores from our previous study [8] where the baseline scores were larger (8.6; SD = 5.4) than in this study (5.4; SD = 3.9). Hence, the sample size was too small to have a significant difference between groups. Additionally, because the baseline K6 scores were already so low in this study, there may have been a floor effect, limiting our ability to determine if family psychoeducation had any effect. In the future, we may also use improved methods to measure the mental health status of patient family members. The second limitation was the short follow-up period. In this study, we ended follow-up at 32 weeks, considering that only 15 months’ follow-up showed beneficial results in the study of Lemmens et al. [15], and a longer follow-up was required. Third limitation of this study was that much of the refusal to participate came from the patients when we asked for their informed consent, because they did not want to bother their family members, or because they already had bad family relationships. Consequently, the families of patients with chronic MDD who had the highest distress may not have participated in the research. Moreover, there were many chronic MDD patients who were living alone because they had already experienced a divorce or were separated from their spouse. Patients with chronic MDD and their spouses may divorce if there is too much distress [3, 4]. This is a distinguishing feature between families of patients with chronic MDD and the families of patients with schizophrenia, who have a similar chronic burden of disease. Because patients with MDD have a relatively late age of onset, their close family members are often spouses.

However, some strengths of this study must be noted. First, this is the first trial to examine the effectiveness of family psychoeducation in improving mental health and maintaining good mental health status in families of patients with chronic MDD. Second, 84% of participants receiving BMP intervention attended all sessions and there was no drop out from the BMP sessions. The lack of any drop-outs may suggest that this intervention is not harmful and potentially a positive experience to participants. Third, our study was designed as an effectiveness study in a daily clinical setting, as evidenced by the broad eligibility criteria for enrollment, use of a variety of clinical staff (9 psychiatrists, 6 nurses, 3 psychiatric social workers, 2 psychologists, and 1 pharmacologist), all of whom (except the pharmacologist) had more than 11 h of standard family psychoeducation training. Fourth, this study was conducted at multiple sites (2 university hospitals, 2 private psychiatric hospitals, and 1 psychiatric clinic). Lastly, statistical analyses were performed by a blinded statistician.

## Conclusion

We failed to demonstrate that BMP in chronic MDD patients and their families had an effect on the mental health status of patient family members. Further studies, with greater patient recruitment and statistical power, are needed to examine the true effect of multifamily psychoeducation on chronic MDD family member mental health. Although family psychoeducation is already an empowering program that educates families on the biology underlying the disease and the best treatment options, it may benefit from additional information on the mental health of family members. Moreover, we must determine how to best provide family psychoeducation for chronic MDD patients and their families.

## Abbreviations

AI: Affective Involvement
AR: Affective Responsiveness
BC: Behavior Control
BDI-II: Beck Depression Inventory
BMP: Brief multifamily psychoeducation
BP: Bodily pain
CM: Communication
DDD: defined daily dose
FAD: The Japanese version of the Family assessment device
FAS: The Japanese version of the Family attitude scale
GF: General Functioning
GH: General Health Perceptions
JNPF: Japanese network of psychoeducation and family support program
J-ZBI_8: The Japanese version of the Zarit Burden Interview short version
K6: Kessler Screening Scale for psychological destress
LS: Latest square
MCS: Mental Component Summary
MDD: Major depressive disorder
MH: Mental Health
MMRM: Mixed model for repeated measures
PCS: Physical Component Summary
PF: Physical Functioning
PS: Problem Solving
RCT: Randomized controlled trial
RL: Roles
RP: Role Physical
SF: Social Functioning
SF-36: The MOS 36-item Short Form Health Survey version 2
VT: Vitality
